# Microparticle and anti-influenza activity in human respiratory secretion

**DOI:** 10.1371/journal.pone.0183717

**Published:** 2017-08-23

**Authors:** Ornpreya Suptawiwat, Kanyarat Ruangrung, Chompunuch Boonarkart, Pilaipan Puthavathana, Kittipong Maneechotesuwan, Komgrid Charngkaew, Nusara Chomanee, Prasert Auewarakul

**Affiliations:** 1 Department of Microbiology, Faculty of Medicine Siriraj Hospital, Mahidol University, Bangkok, Thailand; 2 Center for Research and Innovation, Faculty of Medical Technology, Mahidol University, Nakhorn Pathom, Thailand; 3 Department of Medicine, Faculty of Medicine Siriraj Hospital, Mahidol University, Bangkok, Thailand; 4 Department of Pathology, Faculty of Medicine Siriraj Hospital, Mahidol University, Bangkok, Thailand; Chinese University of Hong Kong, HONG KONG

## Abstract

Respiratory secretions, such as saliva and bronchoalveolar fluid, contain anti-influenza activity. Multiple soluble factors have been described that exert anti-influenza activity and are believed to be responsible for the anti-influenza activity in respiratory secretions. It was previously shown that a bronchial epithelial cell culture could produce exosome-like particles with anti-influenza activity. Whether such extracellular vesicles in respiratory secretions have anti-influenza activity is unknown. Therefore, we characterized bronchoalveolar lavage fluid and found microparticles, which mostly stained positive for epithelial cell markers and both α2,3- and α2,6-linked sialic acid. Microparticles were purified from bronchoalveolar lavage fluid and shown to exhibit anti-influenza activity by a hemagglutination inhibition (HI) assay and a neutralization (NT) assay. In addition, physical binding between influenza virions and microparticles was demonstrated by electron microscopy. These findings indicate that respiratory microparticles containing viral receptors can exert anti-viral activity by probably trapping viral particles. This innate mechanism may play an important role in the defense against respiratory viruses.

## Introduction

Respiratory secretions, such as saliva and bronchoalveolar fluid, are known to contain anti-influenza activity. Several soluble factors in saliva and bronchoalveolar lavage (BAL) fluid have been shown to have in vitro anti-influenza activity. These factors include surfactant protein (SP)-D, SP-A, scavenger receptor gp340, long pentraxin PTX3, L-ficolin, H-ficolin, and serum amyloid P [1–8]. SP-D knock-out mice have been shown to be more susceptible to influenza pneumonia. Most of these factors bind to influenza hemagglutinin (HA) via the lectin-glycan interaction. In this interaction, the anti-viral factors act as either lectin or glycan, whereas HA acts as the other interacting partner. SP-D and SP-A act as lectins and bind to the glycan on the N-linked glycosylation sites on the HA head, whereas HA binds to sialic acid on the glycan of SP-C and SP-B. These respiratory innate antiviral factors may act as the primary defense against influenza and other respiratory viruses.

Extracellular vesicles are formed by membrane budding either on the cell surface as microparticles (MP) or inside the multivesicular body as exosome. While MP is released directly into the extra-cellular space, exosome is released by exocytosis [9]. These membrane-bound vesicles are released from various types of cells as part of their physiologic functions. They play important roles in cell to cell communication using either molecules on their surface or inside the vesicle. MP is believed to be involved in multiple physiological and pathological processes such as coagulation, immune regulation, vascular endothelial function, angiogenesis and oncogenesis [10]. Plasma MP has been proposed as a biomarker for various diseases [11]. MP and exosomes are separated mainly by their size; MP has a size-range of 100–350 nm, whereas exosome is 50–100 nm in diameter. MP is also called microvesicle and ectosome. A previous publication showed that cultured bronchial epithelial cells produced exosome-like particles, which showed anti-influenza activity [12]. However, it was not known whether extra-cellular vesicles with anti-viral activity exist in the respiratory tract in vivo. Therefore, we studied respiratory fluid for the presence of extra-cellular vesicles with anti-influenza activity.

## Materials and methods

### Ethics statement

The human study was approved by the Ethics Committee of the Faculty of Medicine Siriraj Hospital (Siriraj Institutional Review Board), which is in full compliance with the Declaration of Helsinki, the Belmont Report, CIOMS Guidelines and the International Conference on Harmonization in Good Clinical Practice (ICH-GCP). The study was performed under protocol COA No. SI572/2009 ‘‘Sensitivity of Influenza 2009 H1N1 to antiviral factors in bronchoalveolar lavage”. All the human subjects provided written informed consent.

### BAL samples

The BAL samples were collected from 24 patients aged 30–80 years (mean ± SD = 66 ± 11) during January 2009 to October 2015 with a male-to-female ratio of 1:1 who underwent bronchoscopy and bronchoalveolar lavage for the investigation of suspected lung cancer. BAL fluid with 60 ml of sterile normal saline was performed at a non-lesional lung segment. The retrieved fluid was collected and immediately transported on ice to the laboratory. The BAL samples were centrifuged at 1,500 × g at 4°C for 5 minutes. The supernatant was stored frozen in aliquots at -80°C.

### BAL fractionation and MP preparation

The BAL samples were fractionated by Gel filtration Fast protein liquid chromatography (FPLC) (ÄKTA *FPLC*™ system) according to manufacturer’s manual with cold phosphate buffer saline (PBS, pH 7.2) at 4°C using a Hiprep sephacryl S-500 16/60 column (GE Healthcare) with a sample injection volume of 0.5 ml. To purify MP, 500 μl of the BAL samples were centrifuged at 4°C 13,000 × g for an hour. A total of 480 μl of supernatant was collected. The rest of the 20 μl of the pellet was washed by resuspension with 480 μl of 0.25 μm filtered sterile normal saline and centrifuged at 4°C at 13,000 × g for an hour. A total of 400 μl of the supernatant and 100 μl of the pellet were collected. One milliliter and six hundred microliters of the pool of the supernatants collected from the first round of centrifugation were further centrifuged at ultra-high speed at 4°C at 100,000 × g for an hour. A total of 1500 μl of supernatant was collected. The washing process was repeated by resuspending the rest of the 100 μl pellets with 1,500 μl of 0.25 μm filtered sterile normal saline and centrifuged at 4°C at 100,000 × g for an hour. One milliliter and five hundred microliters of the supernatant and the rest of the 100 μl pellets were collected. Each centrifuged sample was tested for anti-viral activity or stained with MP markers.

### Viral strains

The influenza viruses used in this study are from the 2009 pandemic H1N1 (pdmH1N1) influenza viruses (A/Thailand/104/2009 and A/Thailand/MVCU-013/2009) and the seasonal H3N2 influenza virus (A/Thailand/Siriraj-04/2003). All the viruses were propagated for less than 10 passages in Madin-Darby Canine Kidney (MDCK) cells with 1 mML-1-tosylamido-2-phenylethyl chloromethyl ketone (TPCK)-treated trypsin (SIGMA-ALDRICH^®^) in Minimum Essential Medium (MEM) (GIBCO^®^).

### Anti-viral activity assays

To study the antiviral activity, HI and NT were performed. To perform the HI assay, the samples were two-fold serially diluted in PBS, mixed with 4 hemagglutination units of the virus in a 96-well U-shaped microtiter plate, incubated at room temperature for 30 minutes, and mixed with 0.5% Goose red blood cells in PBS at 4°C for 20–30 minutes. The HI titers were read as reciprocal of the highest dilution with complete inhibited hemagglutination. For the NT assay, the BAL was serially diluted in MEM plus 1 mM TPCK-treated trypsin and mixed with 25 TCID50 (50% tissue culture infectious dose) of the virus, incubated at 37°C for an hour and inoculated onto MDCK cells in a 96-well tissue culture plate. Briefly, 3 × 10^4^ cells in 200 μl MEM plus 10% heat-inactivated fetal bovine serum (FBS) (GIBCO^®^) were seeded to each well and incubated at 37°C overnight. After an overnight incubation at 37°C, the tissue culture plate was washed and fixed with 80% cold acetone in 1X PBS; the level of viral infection was measured using an antiviral nucleoprotein monoclonal antibody (Milipore) and a peroxidase-conjugated secondary antibody (Dako). The optical densities (OD) were measured and the NT titers with 50% inhibition of the specific signals were calculated.

For viral replication kinetics in the presence of MPs, MDCK cells at 3 × 10^4^/ 200 μl of MEM plus 10% FBS were seeded to each well of a 96-well tissue culture plate and incubated at 37°C overnight. MEM plus 1 mM TPCK-treated trypsin or 100 μl of H3N2 influenza virus (A/Thailand/Siriraj-04/2003) at 25TCID_50_/100 μl in media was added and incubated at 37°C for an hour. The MDCK cells were washed with media 3 times and added with media or 25 μl of purified MP in 75 μl of media and incubated at 37°C. MDCK cells were observed for cytopathic effect (CPE) at 12, 24, and 36 hours post infection. The entire amount of supernatant in each well was collected and replenished with media with or without MPs at each time point. The supernatants were lysed by heating at 95°C for 5 minutes. A total of 17 μl was used for cDNA synthesis using avian myeloblastosis virus reverse transcriptase (AMV RT) (Promega) and a random hexamer (Promega) in a total volume of 25 μl, and 10 μl of the cDNA was used for real-time PCR using the InfA forward and InfA reverse primers as previously described [13].

The amplification was then performed in a SYBR green dye detection format. The amplification reactions contained 1×RBC ThermOne™ Real-Time PCR Premix with SYBR Green (RBC Bioscience) and 0.4 mM of forward and reverse primer. Melting-curve analyses were performed from 65°C to 95°C. The pHW2000- Matrix -PR8 (A/Puerto Rico/8/1934 H1N1) plasmid was serially diluted, and the threshold cycle of each dilution was determined by RT-PCR for the standard curve.

### MP marker staining

Annexin V-FITC, Annexin V-PE, propidium iodide (PI) and antibodies to CD11b-PE, CD41a-FITC, and CD45-perCP were from BD Biosciences. Fluorescein-labeled *Sambucus nigra* lectin (SNA-FITC) and *Maackia amurensis* lectin I (MALI-FITC) were from Vector Laboratories. Monoclonal antibodies to surfactant protein D (SPD) and keratan sulfate were from Abcam and US Biological Life Sciences, respectively. Goat anti-mouse-Alexa Fluor 488 was from Molecular Probes. Annexin V buffer (0.1 M Hepes (pH 7.4), 1.4 M NaCl, and 25 mM CaCl_2_) was from BD Biosciences.

For the CD marker staining, the BAL samples were stained with 5 μl of the CD marker antibodies and 3 μl of Annexin V FITC or PE in Annexin V buffer at room temperature for 15 minutes. For lectin, SPD and keratan sulfate staining, BAL samples were blocked with 3% bovine serum albumin (BSA; SIGMA-ALDRICH^®^) at room temperature for 30 minutes. Then, after blocking, the samples were incubated with 10 μg of FITC-labeled lectin in phosphate buffer saline (PBS) at room temperature for 30 minutes. For SPD and keratan sulfate staining, the blocked samples were incubated with 1 μg of anti-SPD or anti-keratan in PBS at 37°C for 60 minutes and then stained with goat anti-mouse Alexa Fluor 488 (Molecular Probes) at 37°C for 60 minutes. After the lectin and the secondary antibody staining, the samples were incubated with Annexin V PE in Annexin V buffer at room temperature for 15 minutes. To confirm the MP population by flow cytometry, the stained samples were incubated with 0.1% of Triton X-100 (SIGMA-ALDRICH^®^), which solubilizes MPs without disturbing the protein aggregates [14]. The analysis of MP staining was only performed when the MP signal in the flow cytometry analysis disappeared after the Triton X-100 treatment. Stained MPs were analyzed by CellQuest software (Becton Dickenson) on a FACScalibure flowcytometer (Becton Dickenson). MPs were gated in forward and side scatter plots using 1.33 μm beads (Spherotech) as a size marker.

For apoptotic body staining, the human colon cancer cell line HT-29 (ATCC HTB-38) was treated with hydrogen peroxide in medium-free serum for 24 hours. The cell culture supernatants were collected and centrifuged at 800 × g for 10 minutes to remove the cells and bigger cell debris. The pooled BAL samples and supernatant from the HT-29 cell lines were stained with 3 μl of Annexin V FITC in Annexin V buffer at room temperature for 15 minutes and subsequently stained with 1 μg/ml of propidium iodide (PI) at room temperature for 15 minutes. The stained samples were analyzed by CellQuest software on a FACScalibure flowcytometer.

### Transmission electron microscopy (TEM)

Twenty-five microliters of virus at 10^6^ TCID_50_ and 25 μl of purified MP were mixed. To inhibit neuraminidase activity, 3.75 μl of 20 μM Oseltamivir was added and incubated on a rotator at 4°C for one hour. The suspension was then dropped on a petridish on ice. Grids were coated with the suspension by a floating grid surface on the dropped suspension for 15 minutes. The grids were picked up and fixed with a drop of 5% Glutaraldehyde for 10 minutes and stained with a drop of 1% phosphotungstic acid for 2 minutes on the grid. The grids were dried in a desiccator overnight and observed under TEM.

### Binding of the virus to MPs immobilized on the microtiter plate

The 10 pooled samples of BAL were selected for purified MPs. The MPs were purified from 500 μl of pooled BAL by centrifugation at 13000 × g 4°C for 1 hour. The pellet of the MP was washed once with 500 μl of PBS. Finally, purified MP was resuspended with 50 μl of PBS. For the binding assay, 45 μl of the MPs were pre-treated with 0.005 units of sialidase from *Vibrio Cholerae* (Roche) at 37°C for 1 hour. Either MPs, sialidase treated MPs or MP washed fluid was diluted with coating buffer (0.05M Na_2_CO_3_, 0.05M NaHCO_3_, and pH 9.6) and coated onto a Maxisorb 96-well plate (NUNC^®^) at 37°C for 2 hours. All of the coating mixture was washed 3 times with PBS before blocking with 5% BSA at room temperature for 30 minutes. Next, each well was washed 3 times with PBS. Then, 50 μl of the viruses was added to each well plate and incubation was continued at 37°C for 1 hour. The binding mixture was washed 5 times with PBS before lysis of the complex with 20 μl of 1% Triton X-100. Finally, the RNA in the lysis reaction was mixed together with AMV RT (Promega) for cDNA synthesis. The viral quantity was determined by real-time PCR (RBC ThermOne^™^ Real-time PCR premix with SYBR Green) together with the DNA standard.

### Statistical analysis

The percentages of positive anexinV, SPD and keratan staining were shown in mean±SD. HI/NT titers, % of HI titers and % of MP were shown in geometric mean+SEM. The comparisons of the antiviral activity in BAL fractions, inhibition of viral replication kinetics by MPs, effect of MPs on intracellular viral NP levels, and signal of virus binding to MPs immobilized on microtiter plate were tested by *t*-test. The comparison of MP antiviral activity after sialidase treatment was tested using ANOVA.

## Results

### BAL contain MP with an epithelial origin

**Fig 1:**
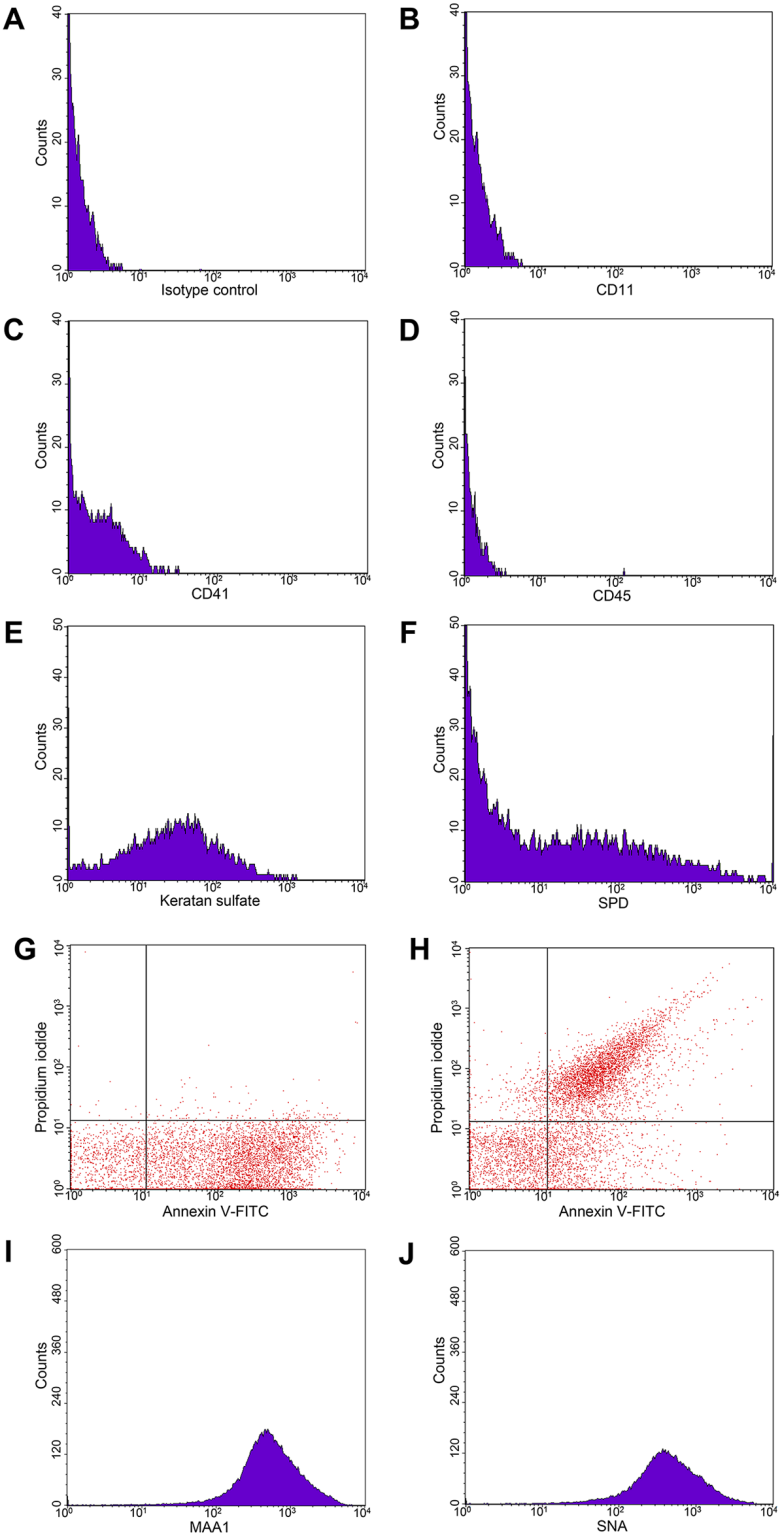
Surface markers of microparticle. BAL was double stained with annexin V and cell surface markers and analyzed by a flow cytometer: isotype control (A), CD11b (B), CD41a (C), CD45 (D), keratan sulfate (E), and SPD (F). MPs were gated using 1.33 μm beads as size markers, and the annexin V-positive gate was analyzed for the expression of the surface markers. To distinguish MPs from apoptotic bodies, BAL was double stained with annexin V and PI (G) using apoptotic bodies from hydrogen peroxide treated HT29 cells as a positive control (H). To detect surface sialic acid on MPs, BAL was double stained with annexin V and lectins. Annexin V-positive gate was analyzed for the expression of SNA (I) and MAA1 (J). The histrogram are representative of ten BAL samples.

BAL has been previously shown to contain MP and exosome. However, their characteristics and origin in the normal physiologic condition is not clear. Since only MP is large enough to be characterized by flow cytometry, we started by characterizing MP in BAL. BAL samples were stained with CD41 (platelet marker), CD45 (leukocyte marker), CD11b (neutrophil marker) and epithelial markers: SPD and keratan sulfate. MPs were identified as particles with positive annexin V staining. Approximately 26.8 ± 8.5 and 56.8 ± 10.7% of MPs were positive for epithelial cell markers, SPD and keratan sulfate, respectively, whereas the platelet, leukocyte and neutrophil markers did not show any significant staining (Fig 1A to 1F). SPD is expressed by alveolar type II pneumocytes and non-ciliated bronchial epithelial (Clara) cells. Keratan sulfate is a proteoglycan previously shown to be abundantly expressed on the apical surface of bronchial epithelial cells. These suggest that BAL MPs were originated from alveolar epithelial and bronchial epithelial cells. Because annexin V can stain both the MP and apoptotic body, we double-stained the BAL samples with annexin V and propidium iodide to distinguish MPs from apoptotic bodies. While a control for the apoptotic body from the supernatant of H_2_O_2_-treated cells showed double-staining for both annexin V and propidium iodide (Fig 1H), BAL samples showed positive staining for only annexin V, which indicated that BAL did not contain a significant amount of apoptotic body, and the annexing V staining in BAL really represented MPs (Fig 1G).

### MP contained surface sialic acid

The binding of influenza A viruses to the host cells requires sialic acids. The α2,3 and α2,6-linked sialic acid is preferred for avian and human influenza virus binding, respectively. To characterize sialic acid on the MP surface, BAL samples were stained by two lectin markers *Maackia amurensis* agglutinin (MAA) and *Sambucus nigra* agglutinin (SNA), which is specific for α2,3 and α2,6-linked sialic acid, respectively. We found that the majority of MPs were positive for MAA and SNA (Fig 1I and 1J). The presence of sialic acid on MPs suggests that MPs might be able to bind influenza virions via its surface sialic acid.

### Purified MP showed anti-influenza activity

**Fig 2:**
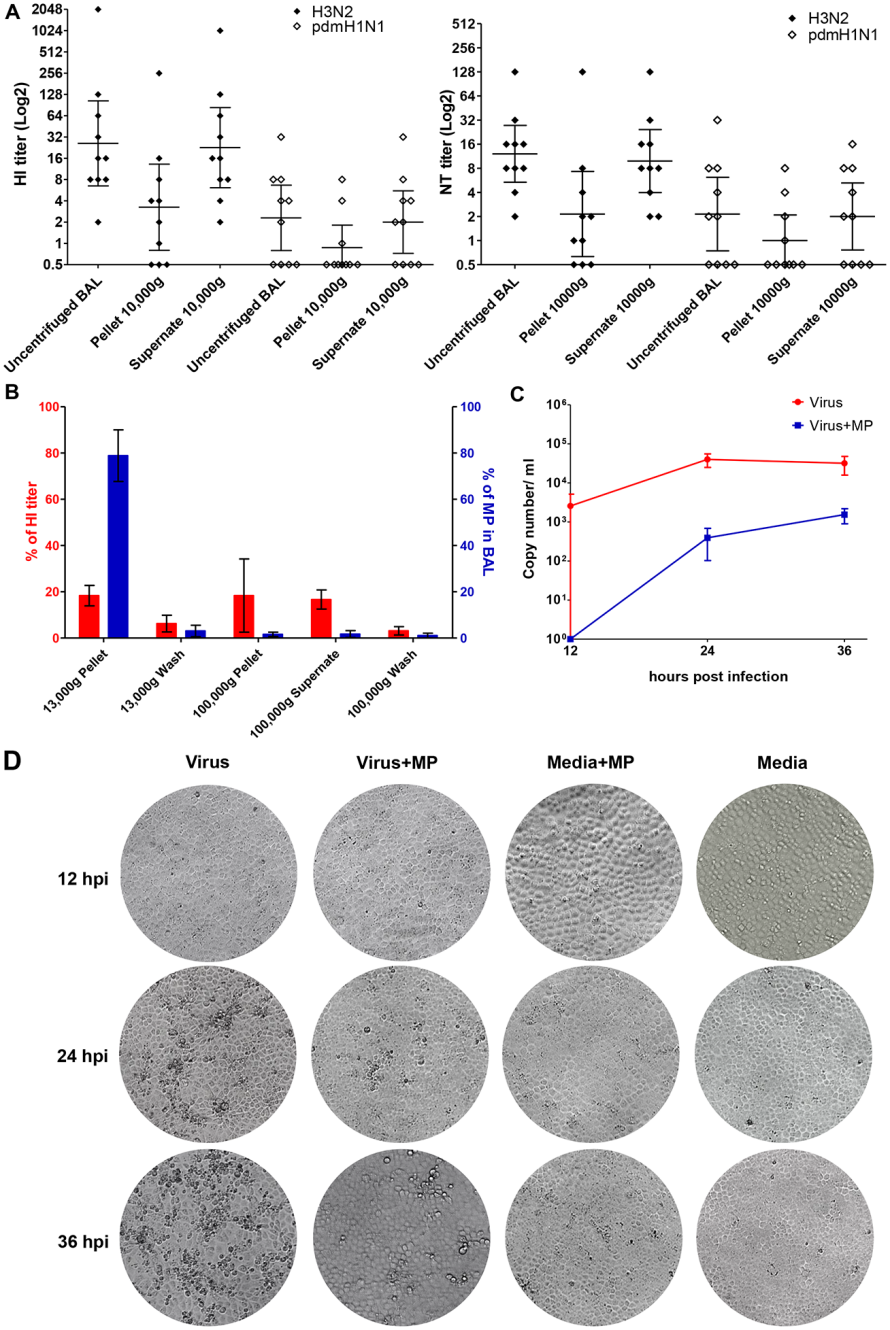
Anti-influenza activity of centrifuged BAL against influenza A virus. (A) HI and NT titers of 10 BAL samples and their MP pellets and supernatant after centrifugation against H3N2 and pandemic H1N1. Both the HI and NT assay were run in duplicate and are shown as the geometric mean±SEM. (B) HI titers and the amount of MP after high (13,000 × g) and ultra-high (100,000 × g) speed centrifugation of BAL samples against H3N2 influenza A virus. The HI assay and flow cytometry were conducted using 10 pooled BAL samples and repeated by 2 other independent sets of 6 pooled BAL samples. HI titers and the amount of MP are shown in the mean±SEM of the percentage compared to un-centrifuged BAL. (C) The kinetics of H3N2 influenza viral replication in MDCK cells with and without MPs was monitored by measuring the viral RNA output in the supernatant after 12, 24, and 36 hours post infection. (D) Protection of the cells against cytopathic effect by MPs was also observed. The experiment was conducted in triplicate and the viral load is shown as the mean±SEM.

Human BAL was shown to have anti-influenza activity by the HI and NT assays [15] and exosome-like particles from cultured human bronchial epithelial cells were shown to have anti-influenza activity [12]. These suggest that BAL MP might contribute to BAL anti-influenza activity. To test whether MP could contribute to BAL anti-influenza activity, human BAL samples were centrifuged at 10,000 × g for an hour to precipitate MP as previously described [16]. The MP pellets and supernatants were tested for anti-influenza activity. The HI and NT activity varied among the BAL samples. This might represent inter-individual variation in the innate mucosal defense or a variation in dilution of BAL fluid by the washing process. Although most of the activity remained in the supernatants after the centrifugation, the MP pellets showed significant HI and NT activity against both H1 and H3 subtypes (Fig 2A). This suggests that both soluble factors and MP contributed to BAL anti-influenza activity. Nevertheless, it was unclear whether all the MPs were precipitated by the centrifugation and whether part of the anti-viral activity in the pellets was due to some leftover soluble factors. To clarify these, washed MP and exosome fractions and soluble fraction were purified from BAL by serial centrifugation. BAL samples were pooled to obtain a sufficient amount for the serial centrifugation. Both MP and exosome pellet fractions at high and ultra-high speed were carefully washed before the HI assay. Each pellet and supernatant fraction was tested for HI activity and for MP count by flow cytometry after annexin V staining. To confirm that the washing steps were sufficient and there were no soluble anti-viral factors contaminated in the pellets, the recentrifugation of the supernatant after each washing was collected and tested for anti-influenza activity. The first centrifugation step precipitated most of the MP from the BAL. The supernatant after ultra-high-speed centrifugation with no significant residual MP was found to contain 18.75±6.25% of the HI activity compared to the uncentrifuged BAL, whereas 15±5% of the HI activity was found in the MP fraction. Little or no significant activity was found in the wash and exosome fraction (Fig 2B). The lack of HI activity in the wash confirmed that the observed activity in the MP fraction was not caused by a contamination of the soluble factors. This also confirmed that both MP and soluble factors contributed to BAL anti-influenza activity. However, the relative activity between MP and soluble factors could not be clearly determined since some of the activity was lost during the repeated centrifugation and washing steps. To further characterize BAL MP antiviral activity, we further studied viral replication kinetics in the presence of purified BAL MPs. In this experiment, culture media was replenished with MPs every 12 hours. MPs clearly reduced the levels of viral replication and the cytopathic effect (Fig 2C and 2D).

**Fig 3:**
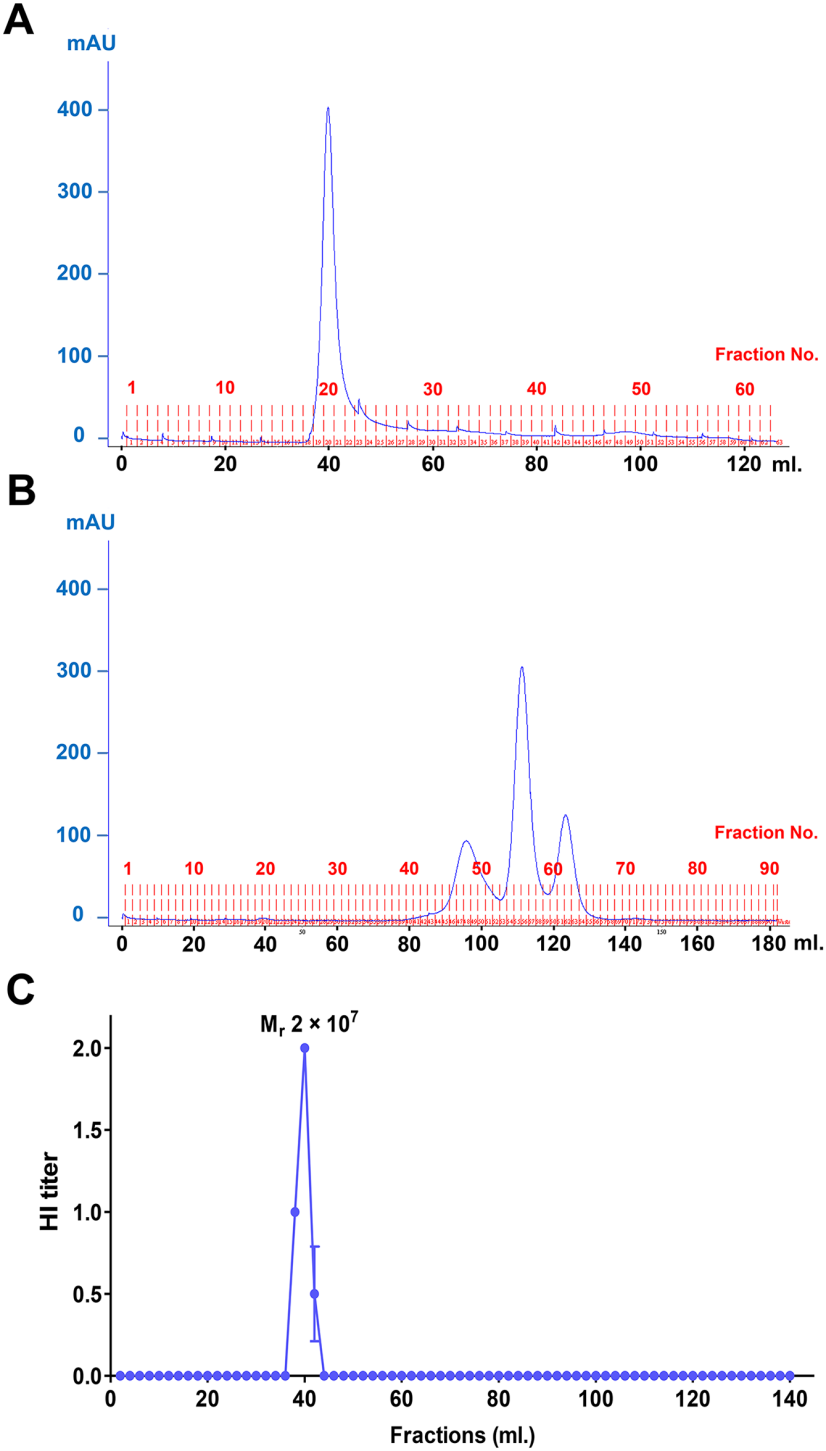
BAL fractionation using FPLC with a Hiprep sephacryl S-500 16/60 column and anti-viral activity in each BAL fraction. (A) The UV (280 nm) absorbance peak as an indicator of the protein concentration of the virus (A/Thailand/MVCU-013/2009) preparation. (B) The UV absorbance peaks of one BAL sample (upper) together with HI titers against pdmH1N1 (A/Thailand/104/2009) from 2 BAL samples (2 sets of fractions) (lower). The HI assay was run in duplicate and repeated by another set of BAL fractions. Each point of the HI titers is shown as the mean±SEM. The molecular mass at the peak of the HI titers was estimated from a dextran molecular weight standard.

The MP anti-influenza activity was further confirmed by FPLC. To separate small particles and soluble proteins, BAL samples were fractionated by size-exclusion using a Hiprep sephacryl S-500 16/60 column. The influenza A virus, which has a size close to MP, was used as a control. A peak of UV absorbance corresponding to virus particles was observed in the void volume (Fig 3A). UV absorbance peaks of the soluble proteins in the BAL sample were observed during 60–130 ml with a predicted size smaller than 25 × 10^3^ Mr. BAL anti-viral activity was observed in fractions immediately after the void volume with a predicted molecular weight of 2 × 10^7^ Mr (Fig 3B). This suggested that the anti-viral factor was almost as large as the virions and should not be soluble proteins. This further supported that BAL MP had anti-influenza activity. It was surprising that no anti-influenza activity was observed in those fractions containing soluble proteins. This suggested that multiple soluble factors contributed to the anti-influenza activity and each of them might have only a little activity, which could not be detected in each fraction.

### MPs bound influenza virions via their surface sialic acid

**Fig 4:**
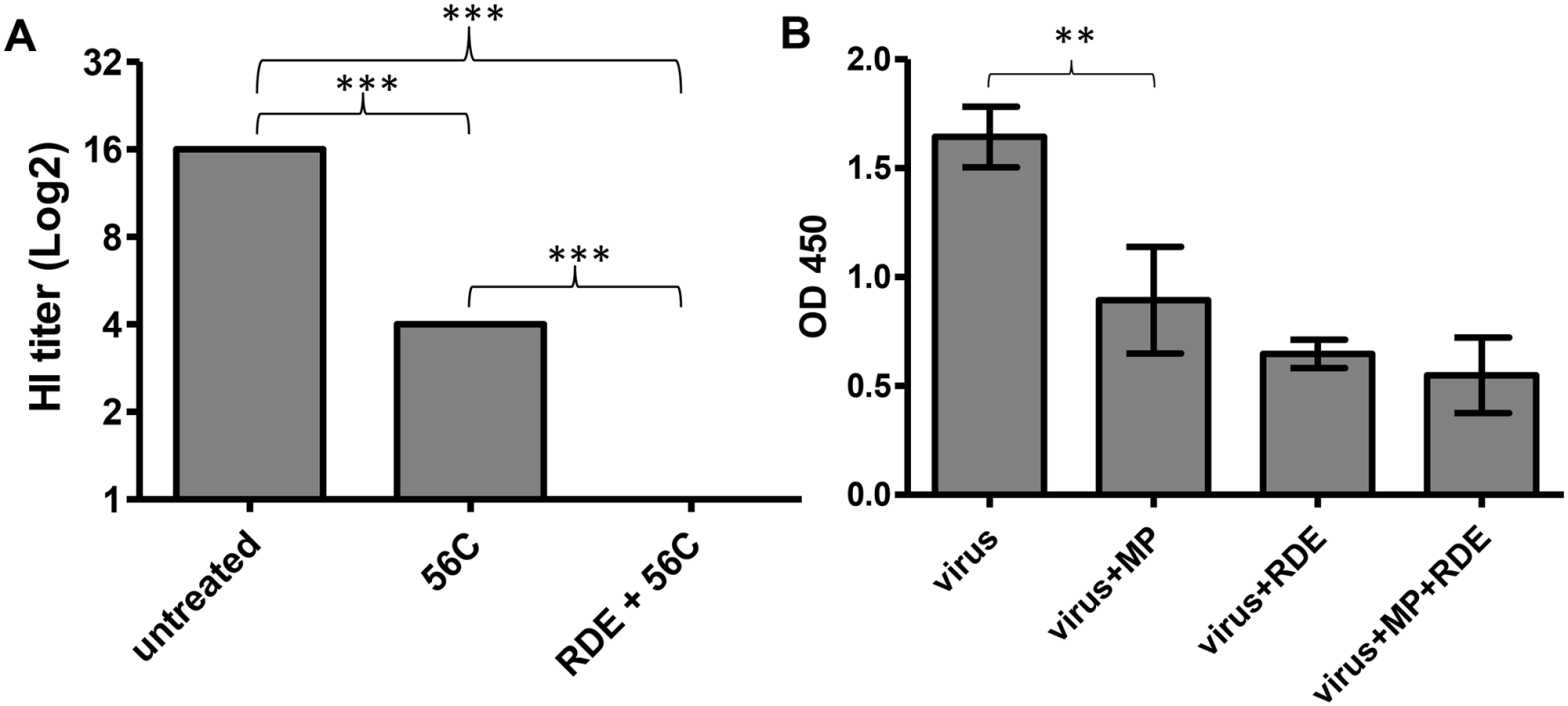
Anti-viral activity by HI against H3N2 influenza A virus of BAL MP preparations with and without the sialidase treatment. (A) Pooled and purified BAL MP was mixed with PBS or RDE at a 1:3 ratio. The samples were rotated at 4°C overnight followed by sialidase inactivation at 56°C for 30 minutes. A sham treated sample was also tested in parallel. The data were derived from 6 pooled BAL samples run in duplicate. The HI titers are shown as the geometric mean±SEM and compared by a *t*-test. The three asterisks (***) represent a significant p-value < 0.0001 by ANOVA. (B) The sialidase treatment did not permanently inactivate the viral infectivity. A/Thailand/Siriraj-04/2003 (H3N2) virus was incubated with either MP or media at 37°C for 1 hour. RDE at a 1:2 ratio was added into either the virus only or the MP+virus and incubated at 4°C overnight. After the incubation, the virus was inoculated into MDCK cells and the viral NP protein was measured after an overnight incubation. The data represented a triplicate result for each condition. The two asterisks (**) represent a significant p-value < 0.01 by the *t*-test.

**Fig 5:**
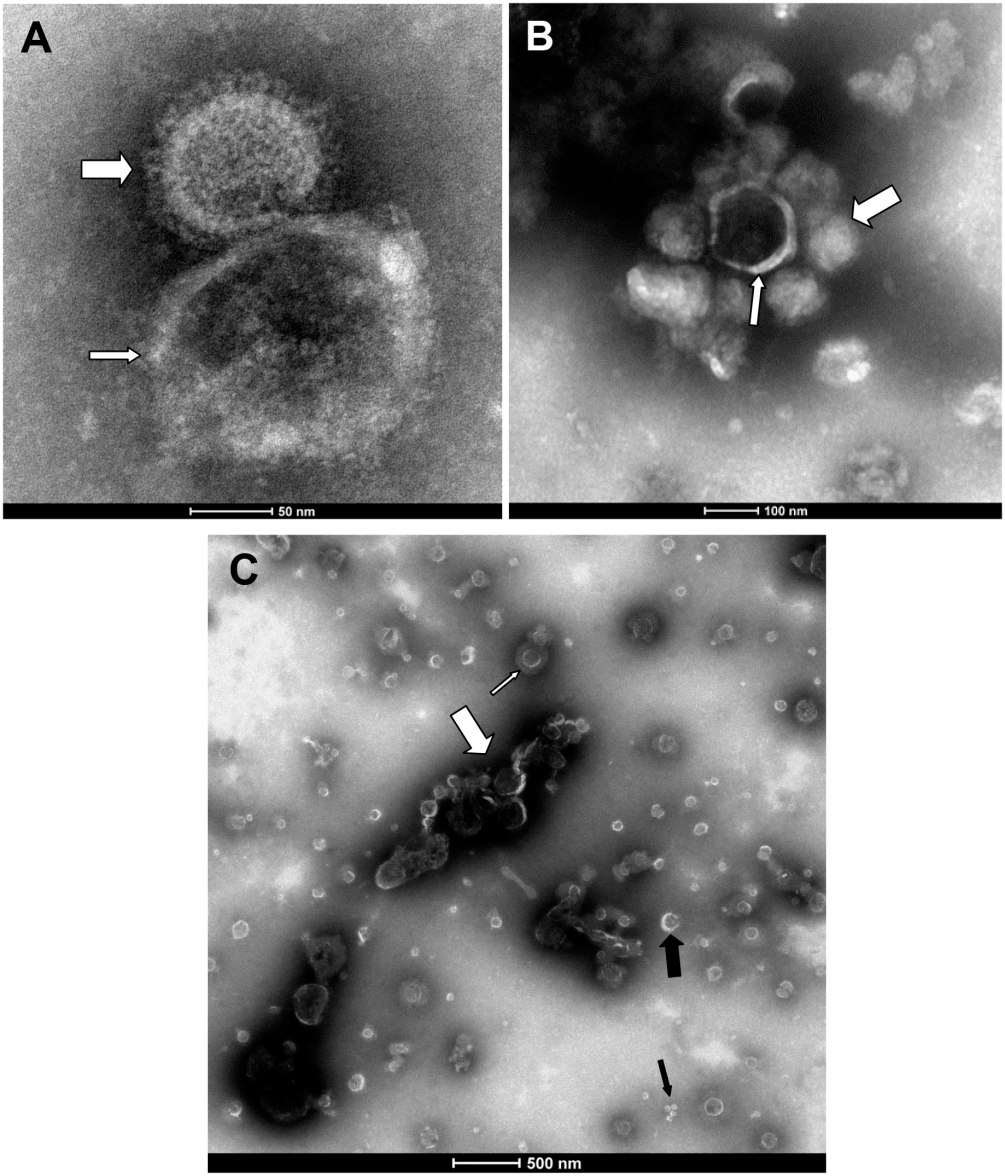
Transmission electron microscopy of the high-speed-centrifuged BAL pellet mixed with the viral preparation. (A) Binding of MP with the smooth surface (narrow arrow) and influenza virion with spikes (A/Thailand/104/2009) (wide arrow); (B) binding of MP (narrow arrow) with multiple virions (wide arrow); and (C) free exosome and MP with smooth membrane appearance shown with narrow and wide black arrows, respectively. The exosome size was usually smaller than 100 nm, whereas the MP was 100–1000 nm in diameter.

**Fig 6:**
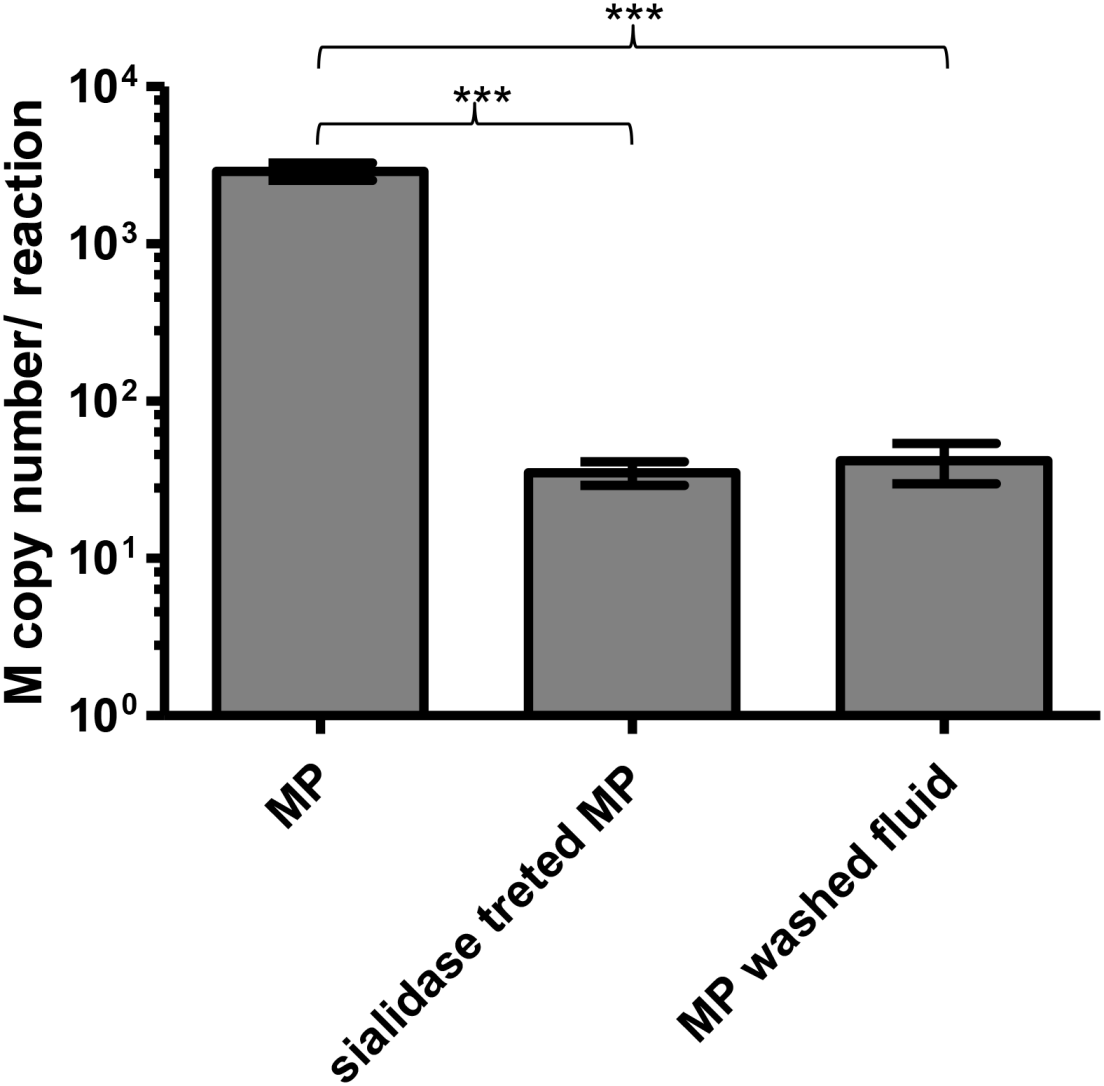
Binding of the virus to MPs immobilized on a microtiter plate. The pooled BAL MPs were adsorbed onto the microtiter plate. After blocking with BSA, the 64HA unit of A/Thailand/MVCU-013/2009 virus was added to bind with the immobilized MPs, and bound virus (after washing) was measured by RT-PCR. MPs pre-treated with sialidase were tested in parallel to demonstrate the requirement of the sialic acid for binding. MP washed fluid was also tested as a negative control. The data represented quadruplicate result for each condition. The three asterisk (***) represent significant p-value < 0.001 by *t*-test.

The presence of sialic acid on the MP surface as shown by lectin staining suggested that MP might exert its antiviral activity by trapping influenza virions using its surface sialic acid. To prove this hypothesis, the MPs were purified, treated with sialidase and receptor destroying enzyme (RDE), and measured for antiviral activity. After the RDE treatment, the MP antiviral activity was completely lost (Fig 4A). Theoretically, the binding of virions to MPs may only trap the virions without inactivating them or cause permanent changes that lead to viral inactivation. To answer this, we treated the virus-MP mixture with RDE after allowing them to bind and inoculated the treated mixture onto MDCK cells. Although RDE itself was toxic to the cells and the infection rate, as measured by intracellular NP staining, was reduced by the RDE treatment, the RDE treatment rescued the viral infection from inhibition by MP and there was no difference in the infection rate with or without MP (Fig 4B). This supports that the trapping of virions by sialic acid on the MP surface as the mechanism of MP antiviral activity and suggests that the trapping did not cause any permanent damage to the virions. To provide further evidence for this mechanism of action, the virus-MP mixture was observed under TEM. Although most of the virions, MP and exosome were found in free particle form, approximately 10% of MP was found bound to virions. Some were found in clumps with multiple virions (Fig 5). To provide more evidence of virion trapping, the MPs were adsorbed onto a microtiter plate, and after blocking, the virus was added to bind with MPs on the plate. After washing, the bound virus was measured by a RT-PCR. MPs clearly showed binding with a significant level of bound virus above the background without MPs, and a sialidase treatment of the MPs eliminated the binding signal, which indicated the requirement of sialic acid for binding (Fig 6).

## Discussion

It was previously shown that human BAL contained MP that originated from various cell types, including respiratory epithelial cells [17–19]. MP has been shown to participate in cell-to-cell communication for various physiological and pathophysiological functions. The communication can be mediated by surface molecules and the contents inside MP such as miRNA [20–21]. Here evidences for another role of MP as an innate mucosal defense are demonstrated. Although our data clearly showed *in vitro* anti-influenza activity of BAL MP, whether and how much this activity contributes to *in vivo* innate respiratory mucosal defense requires further studies. Another limitation of our data is that the BAL samples were from relatively normal subjects, and it is not known whether influenza infection itself can result in a change in the MP content of BAL fluid. BAL MP has been suggested to play roles in lung injury and inflammation and is proposed as a biomarker for lung transplant rejection [17]. Another role of MP in viral infection was described as a viral carrier that can disseminate virus and protect it from the circulating antibody [22–23].

Soluble innate anti-influenza factors are classified as α, β and γ inhibitors. While β inhibitors are Ca++-dependent, heat-labile, and sialidase resistant, α and γ inhibitors are heat-resistant, Ca^++^-independent and sensitive to sialidase. This is because α and γ inhibitors use their sialic acid to bind to influenza HA, whereas β inhibitors act as lectin to bind glycan on HA. We previously showed that a majority of anti-influenza activity in human BAL is heat-resistant, Ca^++^-independent and sialidase-sensitive [15]. This is in agreement with the presence of sialic acid on MP and supports the mechanism of MP anti-influenza activity by using surface sialic acid to trap the influenza virions.

In contrast to the previous publication showing that cultured bronchial epithelial cells produced exosome-like particles with anti-influenza activity [12], we did not observe a significant amount of 50–100 nm particles in human BAL by electron microscopy, and the exosome fraction by ultracentrifugation did not show anti-influenza activity. This suggests that exosome was not an important part of the anti-influenza activity in BAL. Nevertheless, this previous report indicated the possibility of the anti-viral function of extra-cellular vesicles. As MPs are released from the cell surface, they may carry surface molecules of the producing cells, which may include receptors of viruses that can target those cells. This is the case for BAL MP in this study, which contained sialic acid that could bind influenza virus and acted as a decoy receptor to prevent viral infection. Whether this is applicable for mucosal defense against other respiratory viruses and viruses in other types of mucosa requires further studies.
